# Perivascular Spaces Are Associated With CSF Aβ in Cerebral Amyloid Angiopathy but Not in Deep Perforator Arteriopathy

**DOI:** 10.1161/STROKEAHA.125.053794

**Published:** 2026-03-04

**Authors:** Philipp Arndt, Malte Pfister, Sebastian Johannes Müller, Valentina Perosa, Hendrik Mattern, Marc Dörner, Patrick Müller, Sven G. Meuth, Cornelia Garz, Katja Neumann, Jose Bernal, Stefanie Schreiber

**Affiliations:** Department of Neurology (P.A., M.P., C.G., K.N., S.S.), Otto-von-Guericke University, Magdeburg, Germany.; Department of Neuroradiology (S.J.M.), Otto-von-Guericke University, Magdeburg, Germany.; Department of Cardiology (P.M.), Otto-von-Guericke University, Magdeburg, Germany.; German Center for Neurodegenerative Diseases (DZNE) within the Helmholtz Association, Magdeburg (P.A., H.M., M.D., C.G., J.B., S.S.).; J. Philip Kistler Stroke Research Center, Massachusetts General Hospital/Harvard Medical School, Boston (V.P.).; Biomedical Magnetic Resonance, Faculty of Natural Sciences, Otto-von-Guericke University, Magdeburg, Germany (H.M.).; Center for Clinical Brain Sciences, the University of Edinburgh, United Kingdom (J.B., S.S.).; Department of Consultation-Liaison-Psychiatry and Psychosomatic Medicine (M.D.), UniversityHospital Zurich, University of Zurich, Switzerland.; Department of Neurology (M.D.), UniversityHospital Zurich, University of Zurich, Switzerland.; German Center for Mental Health (DZPG), Magdeburg (P.M., S.S.).; Department of Neurology, Heinrich-Heine-University, Düsseldorf, Germany (S.G.M.).; Department of Artificial Intelligence in Biomedical Engineering (AIBE), Friedrich-Alexander UniversitätErlangen-Nürnberg (FAU), Germany (J.B.).

**Keywords:** cerebral amyloid angiopathy, cerebrospinal fluid, magnetic resonance imaging, neuroimaging, siderosis

## Abstract

**BACKGROUND:**

Centrum semiovale perivascular spaces (CSO PVS) are common to different cerebral small vessel disease (CSVD) subtypes, yet their enlargement to the point of visibility on magnetic resonance imaging is thought to occur through distinct mechanisms. In cerebral amyloid angiopathy (CAA), CSO PVS are thought to reflect impaired perivascular Aβ (amyloid-β) drainage, whereas in deep perforator arteriopathy (DPA) they may result from microangiopathy-related arterial stiffening and altered fluid dynamics. The extent to which this can be confirmed in vivo remains unclear.

**METHODS:**

We retrospectively analyzed 186 patients with CSVD and cerebrospinal fluid (CSF) Aβ biomarkers (n=111 probable CAA, n=75 DPA). CSO PVS were counted on axial T2-weighted images. Associations between CSO PVS and CSF biomarkers were assessed via Pearson correlation and multivariable linear regression, including an interaction term between CSF Aβ and CSVD subtype, adjusted for demographics and neuroimaging markers of CSVD.

**RESULTS:**

Patients with higher CSO PVS counts were generally younger, had lower white matter hyperintensity burden, higher basal ganglia PVS counts, and were more frequently affected by cortical superficial siderosis. CSO PVS counts were similar in patients with CAA and DPA. The association between CSF Aβ_42/40_ ratio and CSO PVS burden was observed in patients with CAA, but not in those with DPA (interaction term between CSF Aβ_42/40_ ratio and CAA: β=–0.27; *P*=0.016), independent of demographics and other neuroimaging markers of CSVD. CSF Aβ_40_ showed no association with CSO PVS counts in any model.

**CONCLUSIONS:**

Our findings strengthen the pathophysiological link between CSO PVS and Aβ pathology in CAA but not in DPA. These results extend previous histopathologic and neuroimaging work and underscore the need to interpret CSO PVS in the context of underlying CSVD subtype.

The pathological processes contributing to the enlargement and subsequent visibility of centrum semiovale perivascular spaces (CSO PVS) on magnetic resonance imaging (MRI) remain elusive. In cerebral amyloid angiopathy (CAA), where CSO PVS have supportive diagnostic value,^1,2^ they are thought to reflect impaired perivascular clearance resulting from progressive amyloid-β (Aβ) deposition in cortical and leptomeningeal vessel walls.^3,4^ However, in hypertensive arteriopathy or deep perforator arteriopathy (DPA)—disorders typically associated with hypertensive damage rather than Aβ pathology^5^—CSO PVS are thought to be a result of an impairment of perivascular fluid transport owing to altered hemodynamics (eg, reduced vessel pulsatility and increased arterial stiffness).^6–8^ As such, CSO PVS may thus arise from distinct mechanisms across cerebral small vessel disease (CSVD) subtypes, including microvascular degeneration, blood–brain barrier dysfunction, or other age-related changes.^9^ Whether CSO PVS consistently relates to Aβ pathology or differs between CAA and DPA remains unresolved.

In this study, we examined associations between CSO PVS burden and cerebrospinal fluid (CSF) Aβ biomarkers in a clinical cohort of 186 patients with probable CAA or DPA. Using an interaction model, we tested whether the association between CSO PVS and CSF Aβ differs by CSVD subtype, hypothesizing that CSO PVS reflects impaired Aβ clearance in CAA but not in DPA.

## Methods

### Data Availability

The corresponding author has full access to the data used in this article, and all data are available on reasonable request. See the Supplemental Material for the STROBE guidelines checklist (Strengthening the Reporting of Observational Studies in Epidemiology) required by the journal.

### Study Design and Participants

We retrieved imaging, clinical, and biomarker data from patients with MRI-confirmed hemorrhagic CSVD markers and available CSF Aβ biomarker data from our prospectively maintained CSVD database (2010–2024) at the Department of Neurology, University Hospital Magdeburg.^5^ According to the Standards for Reporting Vascular Changes on Neuroimaging and the Boston criteria v2.0,^1,10^ n=186 patients were classified as probable CAA or DPA. The local ethics committee (No.331 07/2017, addendum 11/2021) approved this study.

### Cerebrospinal Fluid

CSF sampling was performed as part of routine clinical workup in patients with cognitive symptoms, atypical imaging findings, or suspected neurodegenerative copathology. Samples were centrifuged at 4 °C, aliquoted, and stored at −80 °C until analysis. Biomarker levels were determined using ELISA kits (n=133; until 12/2019: Innotest, Innogenetics, Ghent, Belgium) or automated immunoassays (n=53; LUMIPULSE G600 II, Fujirebio Inc, Japan, from 01/2020). To account for methodological variability between assay platforms, CSF Aβ biomarker values were standardized using z-transformation within each assay group, ensuring comparability across methods. Each transformed value reflects the number of SD from the group mean. Thresholds were 0.050 for the Aβ_42/40_ ratio, 70 pg/mL for pTau using ELISA kits, and 0.069 for the Aβ_42/40_ ratio, 56 pg/mL for pTau using immunoassays.

### MRI Acquisition and Analysis

Clinical 3T (n=90, Skyra) or 1.5T (n=96, Sola) MRI scans (Siemens Healthineers) were used to rate cortical superficial siderosis (cSS), microbleeds, white matter hyperintensities (WMH), lacunes, and PVS using Standards for Reporting Vascular Changes on Neuroimaging criteria.^10^ A trained investigator (M.P.), blinded to CSF results, assessed cSS and microbleeds on T2*-weighted GRE, WMH and lacunes on T2-weighted fluid-attenuated inversion recovery, and PVS on axial T2-weighted TSE. Microbleeds and lacunes were rated using the Microbleed Anatomic Rating Scale,^11^ WMH using the Fazekas scale, specific WMH patterns as recently proposed,^12,13^ and cSS using the Boston 2.0 criteria.^1^ CSO and basal ganglia (BG) PVS were quantified as absolute counts on axial T2-weighted images. Continuous counts were chosen over semi-quantitative scores to enhance statistical sensitivity and model variance. A high degree of PVS represents counts of PVS >20 in 1 hemisphere in either BG or CSO. The presence of deep microbleeds defined DPA.

To assess interrater reliability of PVS quantification, n=40 randomly selected MRIs were independently re-rated by a certified senior neuroradiologist (S.J.M.), blinded to the initial ratings. Interrater agreement was good for both regions (CSO: ICC, 0.79 [95% CI, 0.64–0.88]; BG: ICC, 0.75 [95% CI, 0.45–0.88]).

### Statistical Analysis

Results of continuous variables were expressed as median (interquartile range) or mean (SD), and categorical variables were expressed as proportions.

#### Univariate Analysis

Group comparisons were performed in univariate analyses, using the χ^2^ test, 2-sample *t* test, or Mann-Whitney *U* test. Pearson correlation was used to exploratively assess associations between CSO PVS and (1) CSF Aβ_42/40_ ratio as a marker of parenchymal Aβ and (2) CSF Aβ_40_ as a marker of vascular Aβ in CAA and DPA groups separately.

#### Moderation Analysis

To test whether the association between CSF Aβ_42/40_ ratio and CSO PVS counts differed by CSVD subtype (CAA versus DPA), moderation analysis was performed using multivariable linear regression. Models included an interaction term (Aβ_42/40_ ratio×CSVD subtype) and were adjusted for age, sex, lobar microbleeds, cSS, WMH Fazekas scores, and BG PVS counts. CSVD subtype was coded as a binary variable (0=DPA, 1=CAA). All predictors were entered simultaneously based on prior pathophysiological relevance. Statistical significance was defined as *P*<0.05 (SPSS v24).

## Results

Among 186 patients (median age, 75 years [interquartile range, 67–80]; 41% female), 111 were classified as probable CAA and 75 as DPA. Patients’ clinical characteristics included most frequently cognitive impairment (n=101, 54%), history of ischemic stroke (n=61, 33%), gait disturbances (n=55, 30%), cerebral hemorrhage, that is, intracerebral hemorrhage (ICH) or convexity subarachnoid hemorrhage (n=54, 30%; n=33 with probable CAA and n=21 with DPA), and seizure (n=42, 23%). Patients with CAA were significantly older (*P*<0.001) and had lower CSF Aβ_42/40_ ratio levels (*P*<0.001) compared with DPA. CSF Aβ_40_ (*P*=0.297) and CSO PVS counts (*P*=0.413) were, nonetheless, similar between groups (Table 1).

**Table 1.**
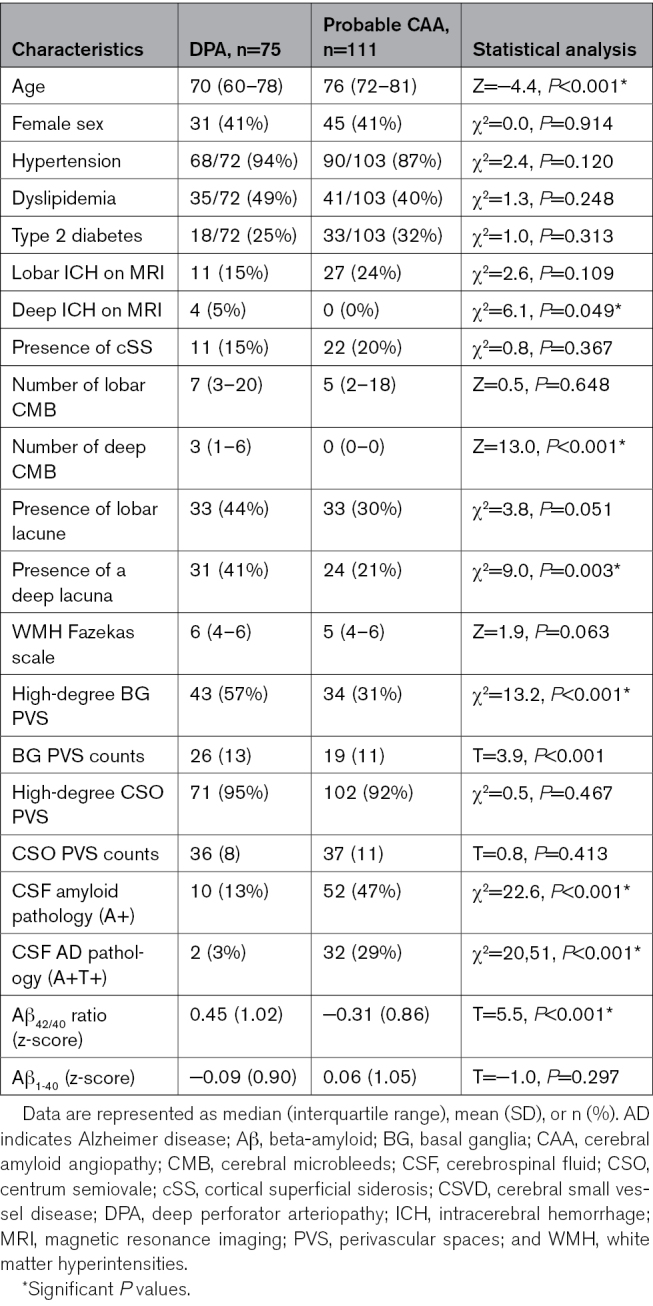
Intergroup Comparison of Patient Characteristics

### Univariate Analysis

Patients with higher CSO PVS counts had lower CSF Aβ_42/40_ ratios (*r*=–0.148, *P*=0.049). This association was primarily driven by patients with CAA (*r*=–0.265, *P*=0.005) and not by those with DPA, according to subsequent group analyses. No association between CSO PVS counts and CSF Aβ_40_ was observed (Figure).

**Figure.**
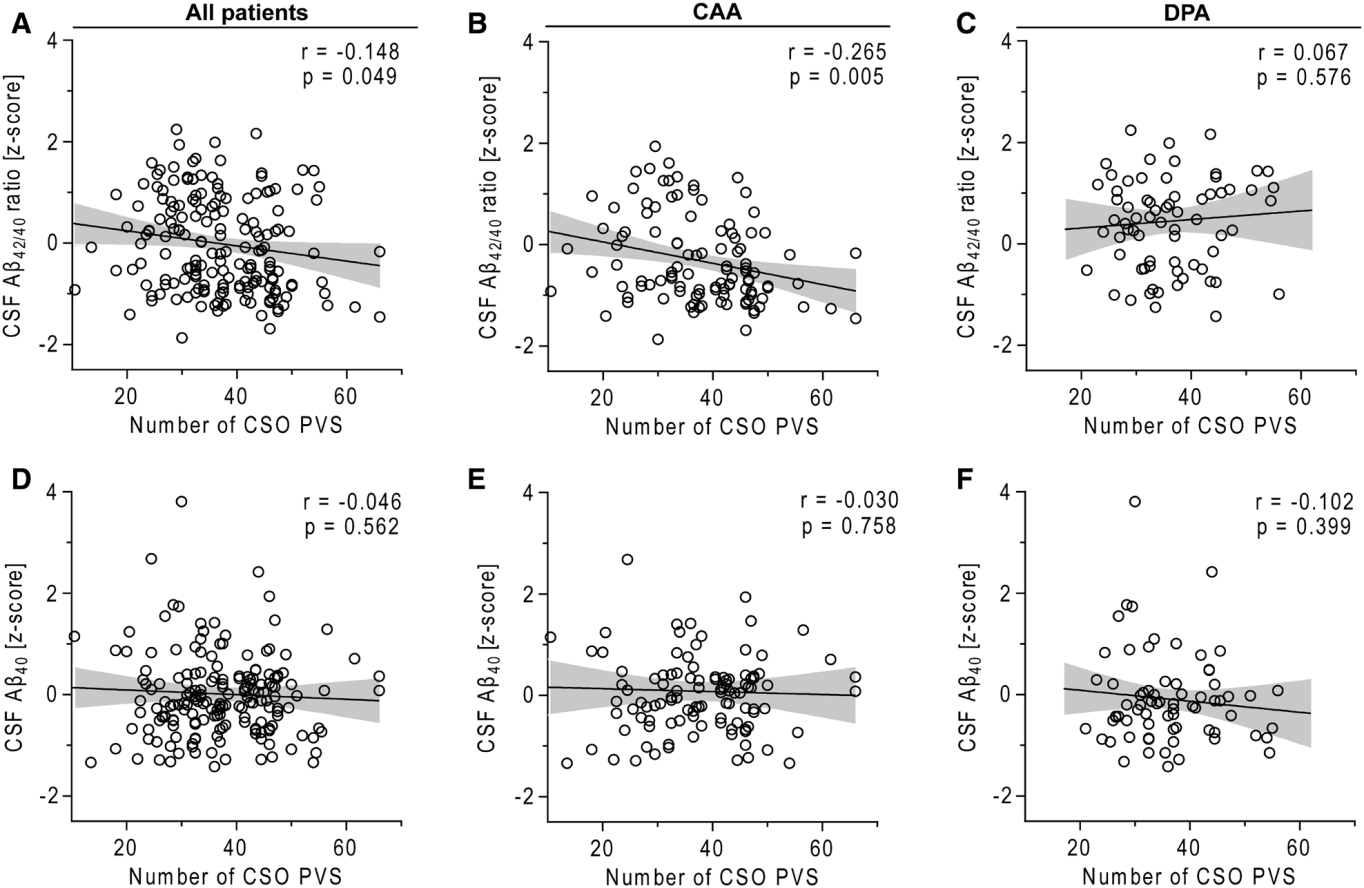
**Scatterplots.** Scatterplots showing associations between centrum semiovale perivascular space counts and cerebrospinal fluid (CSF) amyloid-β biomarkers in cerebral amyloid angiopathy (CAA; **A**–**C**) and deep perforator arteriopathy (DPA; **D**–**F**). In CAA, CSO PVS were negatively correlated with CSF Aβ_42/40_ ratio (**B**) but not Aβ_40_ (**E**). In DPA, no significant associations were observed between CSO PVS and Aβ biomarkers (**C** and **F**). Each dot represents an individual patient. Regression lines represent linear fit with 95% CIs.

### Multivariate and Moderation Analysis

Patients with higher CSO PVS counts were younger (β=–0.26, *P*=0.004), had lower WMH scores (β=–0.24, *P*=0.002) but higher BG PVS counts (β=0.17, *P*=0.027), and were more likely to have cSS (β=0.21, *P*=0.004; Table 2). The association between CSF Aβ_42/40_ ratio and CSO PVS burden was present in patients with CAA but not those with DPA (interaction term β=–0.27, *P*=0.016), independent of age, sex, lobar microbleeds, cSS, WMH burden, BG PVS, and AD pathology. Subgroup analyses revealed that this association was primarily driven by patients without cerebral hemorrhage (n=132, interaction term *P*=0.010) as opposed to those with it (n=54 with ICH or subarachnoid hemorrhage, interaction term *P*=0.962). Replacing Aβ_42/40_ with Aβ_40_ made both the main effect (*P*=0.580) and the interaction with CSVD subtype (*P*=0.930) nonsignificant, suggesting that CSO PVS burden relates specifically to the Aβ_42/40_ ratio.

**Table 2.**
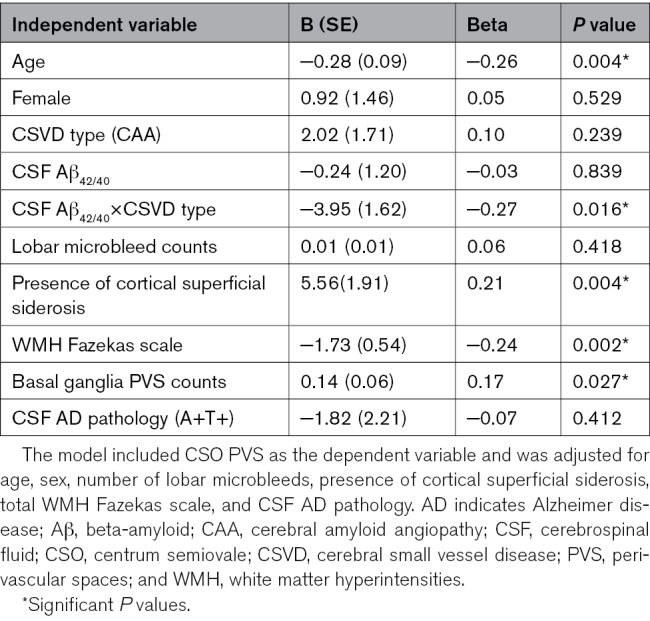
Multivariable Linear Regression Model Examining the Association Between CSO PVS Counts and CSF Aβ Biomarkers in Patients With CSVD

## Discussion

The relationship between CSF Aβ_42/40_ ratio and CSO PVS burden differed by CSVD subtype: lower Aβ_42/40_ levels were linked to higher CSO PVS counts in patients with probable CAA, but not in those with DPA. This association persisted after adjustment for demographics, AD pathology, and other CSVD imaging markers, supporting the notion that CSO PVS may serve as an amyloid-specific structural biomarker of CAA.

Brain autopsies of patients with verified CAA have shown that CSO PVS were associated with overlying cortical CAA severity.^3^ This aligns with our in vivo finding since lower CSF Aβ_42/40_ ratio levels reflect advanced amyloid retention. Interestingly, we observed no association between CSO PVS burden and CSF Aβ_40_. Although Aβ_40_ aggregates in CAA-affected vessels and CSF Aβ_40_ levels are reduced in CAA compared with controls and AD,^14^, its diagnostic performance is limited.^14–16^ This limitation is attributable to substantial interindividual variability and the fact that CSF Aβ_40_ primarily reflects overall Aβ production rather than clearance impairment. Accordingly, Aβ_40_ is mainly used to normalize Aβ_42_ levels, yielding the Aβ_42/40_ ratio as a robust marker of amyloid retention. A contribution of parenchymal plaque pathology cannot be entirely excluded.

Past neuroimaging studies have demonstrated that high-degree CSO PVS are more frequent in CAA-related ICH (46%) compared with deep hemorrhages (19%).^2,17^ Mixed location hemorrhages reflect advanced arteriolosclerotic CSVD, and in mixed location ICH, high-degree CSO PVS (28%) was more frequent compared with deep ICH (15%),^18,19^ showing that CAA-independent mechanisms drive CSO PVS enlargement as well. Interestingly, age and BG PVS were strong predictors. This indicates that there are age-dependent pathomechanisms leading to brain-wide PVS enlargement. A possible interpretation of this result is that the 2 most common sporadic CSVD types (CAA and arteriolosclerosis) both contribute to glymphatic failure and interstitial fluid accumulation.^9^

CSO PVS counts were higher in younger individuals and in those with higher BG PVS counts and lower WMH burden. While WMH and PVS are known to interact and may develop in spatial proximity,^20,21^ our cohort included patients with advanced white matter disease, in whom extensive WMH may limit reliable PVS quantification. This issue has also been noted as a limitation and potential confounder in computational segmentation studies.^22^ The positive association between BG and CSO PVS supports the concept of global mechanisms that affect perivascular fluid drainage across brain regions. Brain atrophy represents an additional potential confounder, as age-related tissue loss may influence the apparent visibility of PVS, and PVS counts are not normalized to tissue volume. Taken together, these findings underscore the need for further studies addressing the interplay between vascular pathology, neurodegeneration, and perivascular space enlargement.

Previous large-scale studies across diverse Alzheimer disease cohorts have reported weak or absent associations between CSO PVS and Aβ biomarkers.^21,23–25^ This inconsistency may be attributed to heterogeneous patient populations and the multifactorial nature of PVS enlargement across disease states. In contrast, smaller PET-based studies focused on CAA have demonstrated a robust link between CSO PVS burden and (vascular) Aβ deposition.^4,26,27^ Our findings validate these associations in a larger, well-defined CAA cohort using CSF biomarkers, supporting the view that in CAA, CSO PVS are a structural correlate of impaired perivascular Aβ clearance, independent of demographics and other neuroimaging markers of CAA. This supports their utility as a non-hemorrhagic imaging biomarker of CAA, complementing other Boston criteria version 2.0 features.

### Limitations

The retrospective and cross-sectional design precludes causal inference. CSF biomarker data were obtained using different assay platforms over time, requiring standardization. The focus of our study—the differential association between CSO PVS burden and CSF Aβ biomarkers across CSVD subtypes—constrained our analysis to individuals with available CSF samples. As CSF testing is not routinely performed in CAA or DPA, our cohort may be enriched for cognitively impaired CSVD patients and may underrepresent individuals presenting with acute cerebral hemorrhage. Further, DPA patients with asymptomatic disease stages or lacunar stroke without microbleeds and without cognitive involvement may follow different pathophysiological trajectories and were underrepresented as well, limiting extrapolation to the full DPA spectrum. Replication in unselected or population-based CAA and DPA cohorts should therefore be undertaken in the future. In addition, CSO PVS were assessed via visual counts rather than automated volumetric segmentation, which may limit reproducibility. MRI scans were acquired across clinically diverse protocols and platforms, which could introduce variability in image resolution and contrast. However, as PVS were quantified on the single axial T2-weighted slice demonstrating the highest PVS burden, the impact of variability of slice thickness is reduced. Consistent rating procedures, good interrater reliability, and blinded assessments further mitigate these concerns.

### Conclusions

Our findings reinforce that CSO PVS are not uniform imaging markers of small vessel disease but exhibit subtype-specific relationships with amyloid pathology. The association with CSF Aβ_42/40_ ratio was specific to CAA and absent in DPA, supporting differential underlying pathophysiological mechanisms. These results extend previous histopathologic and neuroimaging work and underscore the need to interpret CSO PVS in the context of underlying CSVD subtype. Although not causal, the observed associations suggest that CSO PVS may hold distinct diagnostic and pathophysiological relevance depending on the vascular phenotype. Future studies using automated PVS quantification, longitudinal follow-up, and structural imaging metrics such as atrophy will help define their utility as imaging biomarkers across the CSVD spectrum.

## ARTICLE INFORMATION

### Author Contributions

Dr Schreiber contributed to the conception and design of the study; Drs S.J. Müller, Pfister, P. Müller, and Garz contributed to the acquisition and analysis of data; Drs Arndt, Bernal, and Schreiber contributed to drafting the text; Dr Arndt contributed to preparing the figures and tables. All the authors contributed to a critical review of the article.

### Sources of Funding

### Disclosures

Prof Meuth reports compensation for other services from Alexion Pharmaceuticals, Janssen Pharmaceuticals, Ono Pharmaceuticals, Sanofi Genzyme, Hexal AG, Amicus Therapeutics, Springer, BioNTech, UCB, Stada, Teva Pharmaceutical Industries, Celgene, Bayer Healthcare, Mylan, Novartis, Almirall, Merck, Roche, Biogen, and Viatris. The other authors report no conflicts.

## Supplementary Material


